# Asymmetries of the Arcuate Fasciculus in Monozygotic Twins: Genetic and Nongenetic Influences

**DOI:** 10.1371/journal.pone.0052315

**Published:** 2013-01-02

**Authors:** Isabelle S. Häberling, Gjurgjica Badzakova-Trajkov, Michael C. Corballis

**Affiliations:** 1 Research Centre for Cognitive Neuroscience and Department of Psychology, The University of Auckland, Auckland, New Zealand; Johns Hopkins School of Medicine, United States of America

## Abstract

We assessed cerebral asymmetry for language in 35 monozygotic twin pairs. Using DTI, we reconstructed the arcuate fasciculus in each twin. Among the male twins, right-handed pairs showed greater left-sided asymmetry of connectivity in the arcuate fasciculus than did those with discordant handedness, and within the discordant group the right-handers had greater left-sided volume asymmetry of the arcuate fasciculus than did their left-handed co-twins. There were no such effects in the female twins. Cerebral asymmetry for language showed more consistent results, with the more left-cerebrally dominant twins also showing more leftward asymmetry of high anisotropic fibers in the arcuate fasciculus, a result applying equally to female as to male twins. Reversals of arcuate fasciculus asymmetry were restricted to pairs discordant for language dominance, with the left-cerebrally dominant twins showing leftward and the right-cerebrally dominant twins rightward asymmetry of anisotropic diffusion in the arcuate fasciculus. Because monozygotic twin pairs share the same genotype, our results indicate a strong nongenetic component in arcuate fasciculus asymmetry, particularly in those discordant for cerebral asymmetry.

## Introduction

Broca’s and Wernicke’s area are dorsally connected via the arcuate fasciculus, which transfers linguistic information important for phonological and syntactic processing of words and sentences [1], [2]. The strength of the anatomical connections in the arcuate fasciculus might therefore play an important role in the asymmetric distribution of the language network and in the maintenance of hemispheric specialization. Imaging techniques such as diffusion tensor imaging (DTI) allow the study of white matter tracts in vivo by reconstructing their anatomical locations and quantifying the underlying white matter coherence. There is some evidence that the arcuate fasciculus might be asymmetric in showing a larger volume [3], [4] or a higher fiber density [5] in the left hemisphere than the right. Studies investigating white matter coherence by extracting FA values, an indirect measure of myelination and/or axonal density in white matter [6], generally show higher FA values in the left arcuate fasciculus than in the right, using different methods such as whole brain voxel-based statistics [7], or reconstruction by tractography [4].

The functional significance of this leftward asymmetry remains somewhat unknown, given that several studies correlating arcuate fasciculus asymmetry with functional language lateralization have yielded conflicting results. One study showed the asymmetry of the fiber density of the arcuate fasciculus to be correlated with functional language lateralization in right-handers in Wernicke’s area but not in Broca’s area, but there was no such correlation in left-handers [5]. In a similar study, though, a structure-function covariation was evident only in consistent left-handers [8]. Another study showed left-lateralization of the FA values to be positively correlated with left-hemispheric language dominance [4], but this study included only right-handers. In a study of epilepsy patients in which language lateralization was established using sodium amobarbital injection, considered the gold standard in determining functional language dominance, asymmetry of arcuate fasciculus fibers with high anisotropy values determined language lateralization correctly in 16 of the 19 left-cerebrally dominant and in 3 of the 4 right-cerebrally dominant patients, suggesting moderate correlation at best [9].

The conflicting results obtained in previous studies might be partly due to other factors such as gender and handedness influencing arcuate fasciculus asymmetry, obscuring relations to language dominance. One way to reduce the interindividual variability is to study monozygotic (MZ) twins, who share the same set of genes and are therefore perfectly matched for age, gender and genotype. Moreover, some 20–25% of identical twins are of opposite handedness [10], [11] and some twins also show reversed cerebral asymmetry for language [12], [13], suggesting sufficient variation to test for correlation with arcuate fasciculus asymmetry. In particular, any variation within pairs of twins would indicate a nongenetic source.

We used a verbal fluency paradigm during fMRI to measure functional language dominance in 35 monozygotic twin pairs of which 18 pairs were of discordant handedness. All twins also underwent a DTI sequence and the arcuate fasciculus in each twin was reconstructed. This enabled us to compare the arcuate fasciculus in two perfectly matched individuals but with different strength and/or direction of hemispheric language dominance. If arcuate fasciculus asymmetry is related to functional language dominance we might expect that the more left-hemispheric twin would show a more left-hemispheric asymmetric arcuate fasciculus than his or her less left-hemispheric dominant co-twin.

## Materials and Methods

### Subjects

Ethics approval was obtained by the Human Ethics Participants Committee at the University of Auckland, New Zealand, and all subjects gave written consent prior to the study. 35 pairs of monozygotic twins (14 male/male, mean age = 23.5, SD = 7.8, 21 female/female; mean age = 25.5, SD = 9.4) took part in the study. Each twin member filled out a short zygosity questionnaire consisting of questions regarding their physical resemblance, the difficulty for family and friends to tell them apart, and their hair and eye color in childhood [14]. In 7 twin pairs, where zygosity could not be established beyond doubt based on the answers and the physical appearance, additional DNA testing was performed. DNA was extracted from mouth swab samples and the individuals were genetically typed using the multiplex PCR kit AmpFISTR Identifiler (Applied Biosystems). The kit compares the STR (short tandem repeat) profiles on 15 highly polymorphic loci. Twins were considered to be monozygotic when no differences in the 15 loci were detected [15].

Based on writing hand, 16 pairs were both right-handed (RR pairs; 7 male/male; 9 female/female; mean age = 22.6, SD = 7.2), 1 pair were both left-handed (LL pair; 1 female/female; age = 21), and 18 pairs were of opposite handedness (RL pairs; 7 male/male; 11 female/female, mean age = 26.8, SD = 10.0). Twin pairs with discordant handedness were deliberately over-represented in the study. Each twin also filled out a handedness inventory, in which they indicated the preferred hand in 12 activities: writing, throwing a ball, holding a racquet, lighting a match, cutting with scissors, threading a needle, sweeping with a broom (top hand), shoveling, dealing cards, hammering, holding a toothbrush, unscrewing a lid [11]. They gave two ticks for the preferred hand or one tick for each hand if there was no preference. A laterality index (LI) was then calculated from the formula 100×(R−L)/(L+R) where R and L represent the number of ticks for the right and left hand, respectively. Although the primary criteria for handedness was the writing hand, as has been suggested by McManus [16], all left-handed twin members had LIs <40 and all right-handed twin members had LIs >50, resulting in a classification scheme similar to that used in previous research [17].

### Word Generation Task

To assess language dominance, all participants undertook a word generation task adapted from the Controlled Oral Word Association test [18] during fMRI. Participants were asked to silently generate as many words as possible starting with a designated letter (F, A, S, B, and M) which were randomly and centrally projected onto a screen (in Courier New black font, size 50). They were instructed not to use proper names or the same words with different endings. The letters were presented for 30 s, followed by a 30 s baseline period that consisted of a black cross, resulting in an acquisition time of 5 min. Prior to the experiment, all participants completed a comparable short version of the task, naming the words overtly, in order to obtain a behavioural performance measure.

### Image Acquisition and Processing

MRI scanning was performed on a 1.5-T Siemens Avanto scanner (Erlangen, Germany). A T1-weighted structural image was acquired using a 3-D MP-RAGE sequence with 176 axial slices parallel to the AC-PC line, ensuring whole brain coverage. The following parameters were used: TR = 11 ms; TE = 4.94 ms; flip angle: 158; FOV: 256×256 mm^2^. Slice thickness was 1 mm and the interslice gap 0, resulting in isotropic voxel resolution of 1×1×1 mm. For the diffusion weighted images a single-shot spin echo sequence along 30 diffusion gradient directions with a b0 of 1000 s/mm^2^ was used with the following parameters: TR = 6601 ms, TE = 101 ms, FOV = 230 mm; in-plane resolution: 1.8×1.8 mm; slice thickness 3 mm. In addition, one image without diffusion weighting was acquired. The sequence was repeated twice resulting in an acquisition time of approximately 7 min. The EPI acquisition had the following parameters: TR = 2500 ms; TE = 50 ms; flip angle = 90; FOV = 192×192; matrix size: 64×64; 29 slices parallel to the AC-PC line; slice thickness: 3 mm; interslice gap: 25% = 0.8 mm.

### Functional Imaging Processing and Analyses

The functional images were analysed using SPM5 software (Wellcome Department of Imaging Neuroscience, London, UK; www.fil.ion.ucl.ac.uk). First, the standard pre-processing steps (realignment, coregistration, normalization and smoothing) were applied. The functional scans were realigned to the first image of the session and the mean of the functional volumes was calculated. The T1-weighted structural image was then coregistered to the previously obtained mean of the functional volumes. Then, all images were normalized into standardized stereotactic space (MNI, Montreal Neurological Institute) and spatially smoothed with an anisotropic Gaussian filter of 9×9×9 mm of full-width at half maximum (FWHM). For each participant, the functional volumes were subjected to a fixed-effects analysis using the general linear model that was applied at each voxel across the whole brain. The model was set up as a box-car function with the two alternate conditions letter vs. baseline. The resulting function was convolved with a canonical haemodynamic response function and movement regressors were also included in the model. For the group analysis, a second-level random effects analysis was performed by applying a one sample t-test to the contrast images of the first-level analyses. A family-wise error (FWE) correction was applied at p<0.05 with a contiguity threshold of 10 voxels.

To establish language dominance, laterality indices were calculated by comparing the activity between the left and right Broca’s region using the formula LI = (L−R)/(L+R), where L and R represent activations in the left and right hemisphere, respectively. The laterality toolbox available on the SPM website was used to calculate the laterality indices [19]. It applies a bootstrap algorithm to calculate about 10,000 indices at different thresholds yielding a robust mean LI ranging between -1 for extreme right to +1 for extreme left lateralization. For each participant, the weighted mean LI for Broca’s area was computed. Broca’s area was defined using the WFU Pick Atlas toolbox [20] and included Brodmann areas 44 and 45. The masks were smoothed with a 6 mm Gaussian filter to control for inter-individual variability. In addition, the toolbox integrates a mask weighting factor that represents the relation of the volumes of the masks on the left and on the right to rule out influences of different mask sizes. A positive index within the ROI corresponded to a left-hemispheric dominance (LI >0.1) and a negative index to a right-hemispheric dominance (LI<−0.1) [21]. Due to the small size of the bilateral group of 2 subjects (LIs between −0.1 and +0.1), these were assigned to the right-hemispheric group since their hemispheric dominance pattern is also atypical in nature.

Although there has been some question as to the reliability of fMRI-based laterality measures [22], classification of language lateralization based on this task has yielded results consistent with the Wada test, which is thought to be the “gold standard” for measuring hemispheric dominance [23], [24], and has also yielded results consistent with fTCD [25]. Moreover, the test-retest reliability of lateralization has been shown to be high [21], [26].

**Figure 1:**
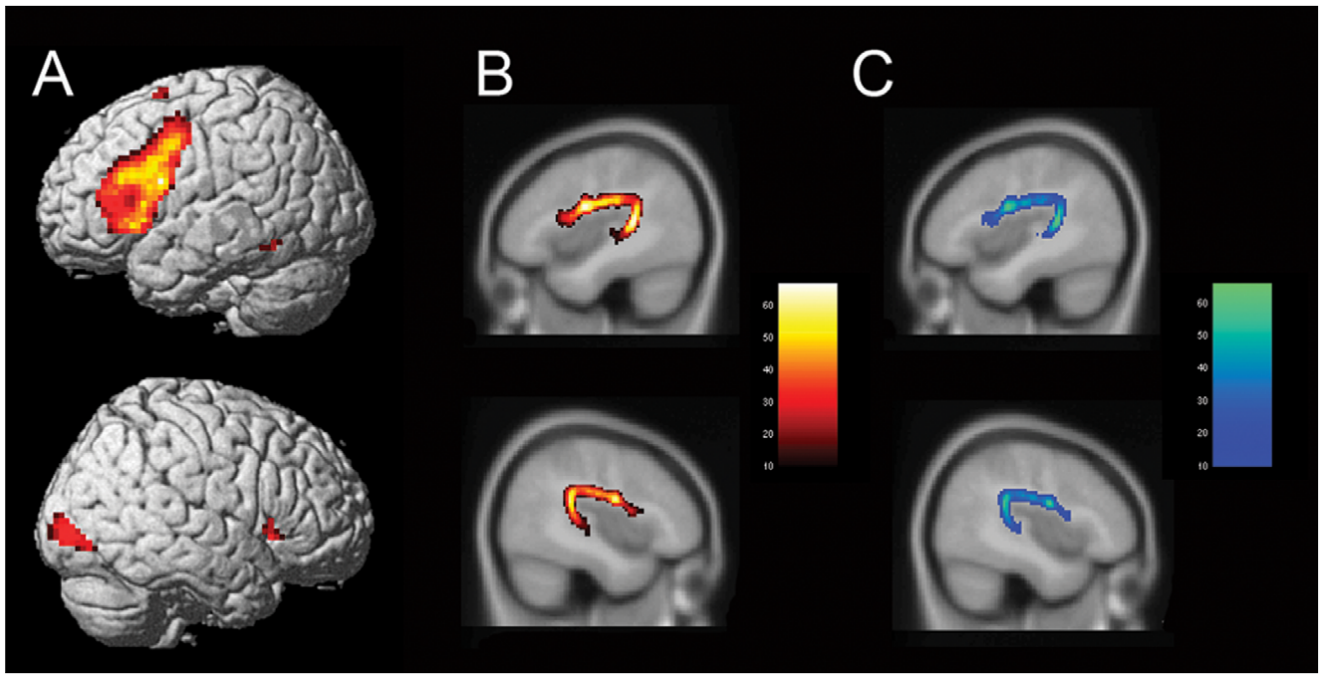
Brain activity and the arcuate fasciculus. Averaged brain activity during word generation task (**A**) and reconstruction of the arcuate fasciculus on the **B**) 1% threshold and **C**) 2% threshold, respectively. Colour bars represent the number of subjects showing arcuate fasciculus fibers in that voxel.

The word generation paradigm induced significant leftward activations in the inferior frontal gyrus, including pars opercularis and pars triangularis, insula, precentral gyrus, SMA, and inferior temporal gyrus (see Figure 1 A). Additional right-hemispheric clusters were observed in the inferior and middle occipital gyrus. Overall, a one-sample t-test revealed that the laterality indices were significantly leftward asymmetric in Broca’s area (M = 0.61, SE = 0.051, t(69) = 12.1, p<0.001).

#### DTI preprocessing and analysis

The tractography of the arcuate fasciculus was carried out using the software program FSL (http://www.fmrib.ox.ac.uk/fsl/). The diffusion data were corrected for eddy currents and head motion and the two acquisitions were averaged to improve signal-to-noise ratio. Diffusion tensors were fitted at each voxel and FA maps were generated. Then, the probability distribution of fiber direction at each voxel was calculated using previously described methods [27]. A seed mask was drawn on a coronal section at the level of the precentral sulcus by identifying voxels in which the principal diffusion direction was anterior-to-posterior (voxels colored green) [28]. For each participant, probabilistic tractography was run from this seed mask by drawing 5000 random samples with a steplength of 0.5 mm and a curvature threshold of 0.2. In order to separate arcuate fasciculus tracts from erroneous tracts, a further two ROI approach was used by defining a posterior ROI comprising fibers reaching the white matter beneath the middle and superior temporal gyrus, and an anterior ROI containing fibers in the white matter beneath the inferior frontal gyrus. Then, the arcuate fasciculus was tracked twice, once from the anterior to the posterior ROI, and once from the posterior to the anterior ROI. The resulting tracts were visually inspected for consistency with the known anatomy. Given inconsistent reports of the exact course of the arcuate fasciculus [29], [30], [31], [32], [33], fibers were only tracked between the two ROIs, excluding indirect pathways connecting the two areas.

Voxels with FA <0.2 were considered grey matter and excluded from the analysis [34]. The two pathways were then added together and were thresholded at 1% and 2%, respectively of the total streamlines connecting the target ROIs. The results were analyzed on two different thresholds given that laterality indices for FA values increase over higher thresholds [4] and to ensure that the results were not threshold dependent. To extract the DTI parameters, namely fractional anisotropy (FA), mean diffusivity (MD), axial diffusivity (AD), and radial diffusivity (RD), the tracts were binarized and multiplied with the individual DTI images to get the mean value of the parameter over the whole tract. To extract the volume of the tracts that were corrected for brain size, all images were normalized to MNI standard space and the normalized volumes were calculated [4]. In 3 of 70 subjects the arcuate fasciculus was intractable in one of the hemispheres and the pairs to which they belonged were excluded from analysis. Previous studies have shown that normal variation in this structure results sometime in intractability [8], [35], [36].

#### Statistical analyses

In order to detect potential differences in the mean values between different groupings of twins, twin pair was treated as a random factor, while group and twin gender were entered as between-twin factors into the analysis of variance. Subjects (individual twins) were treated as nested within each twin pair. All post-hoc tests were performed with a Bonferroni correction for multiple comparisons.

## Results

We established cerebral asymmetry for language by computing laterality indices (LI) based on activity in Broca’s area, with an LI >0.1 reflecting left-hemispheric language dominance (see Fig. 1A). A classification of the twin pairs according to handedness, language dominance and gender is shown in Table 1. Overall, 24 pairs were concordant for left-hemispheric language dominance, 7 pairs were discordant for language dominance and 1 pair was concordant for atypical language dominance. We then carried out tractography of the arcuate fasciculus in each twin (Fig. 1B) and assessed connectivity by calculating the normalized tract volumes and the mean values of the diffusion parameters (FA, MD, AD, and RD) over the whole tract. For all measurements, we calculated asymmetry indices (AI) according to the formula AI_DTI_ = (DTI_L_ - DTI_R_)/(DTI_L_+DTI_R_), where DTI represents the specific diffusion parameter.

**Table 1:** Concordance for handedness and language dominance in male and female twin pairs.

		Language dominance			Total
	Handedness	both left	discordant	both atypical	
Male	both right	6(1)	–	–	7
	discordant	4	2(1)	–	7
	both left	–	–	–	
female	both right	6(1)	1	1	9
	discordant	8	3	–	11
	both left	–	1	–	1
Total		26	8	1	35

Twin pairs who were excluded from the analysis due to the intractability of the arcuate fasciculus are shown in brackets.

### Handedness Effects

We first tested the effect of handedness on diffusion asymmetry by assigning the pairs to two groups, one in which both were right-handed (RR group; N = 14) and one in which the pairs were of opposite handedness (RL group; N = 17). One pair was excluded because both members were left-handed. In the RL group, twins were sorted so that the first twin in each pair was the right-handed twin member.

**Figure 2:**
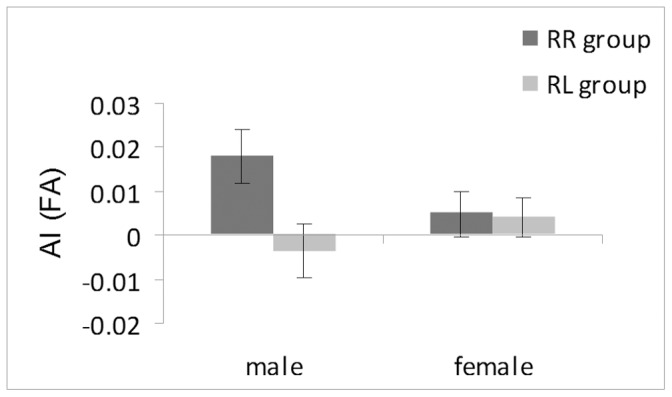
Handedness. Asymmetry indices (AI) for the FA values of the arcuate fasciculus according to handedness and gender are depicted. Male twin pairs with discordant handedness showed significantly lower AI_FA_ than right-handed male twin pairs whereas the same effect was not observed for the female twin pairs.

Analysis of variance showed an interaction between gender and handedness group (F_(1,27) = _4.31, p = 0.048), as shown in Figure 2. For males, the RR group showed a higher AI_FA_ index (mean = 0.018) than the RL group (mean = −0.004, p = 0.017), but for females there was no significant difference (means = 0.004 and 0.005, respectively, p = 0.888). Within the RL group, it was possible to compare the left-handed twins with their right-handed co-twins, with left-handers (mean = −0.004) not significantly different from the right-handers (mean = 0.005, p = 0.547).

For MD asymmetry we found a three-way interaction between handedness group, gender and threshold (F_(1,27)_ = 4.296, p = 0.048). Among the male twin pairs, the RR group showed significantly lower values than the RL group across both thresholds (RR group, M = −0.012, SE = 0.005; RL group, M = 0.007, SE = 0.005), whereas the female twin pairs did not significantly differ (RR group, M = −0.004, SE = 0.004; RL group, M = −0.003, SE = 0.004). Additionally, among the right-handed male twin pairs, the asymmetry was less pronounced on the 1% threshold (M = −0.011, SE = 0.005) than on the 2% threshold (M = −0.013, SE = 0.005). Analysis of RD and AD asymmetry revealed no significant effect of handedness or gender.

Over the whole sample, the volume of the arcuate fasciculus was leftward asymmetric across both thresholds (1%: T(66) = 7.678, p<0.001; 2%: T(66) = 7.172, p<0.001). In addition, we found an interaction between handedness and gender that was restricted to the RL group (F_(1,27) = _4.92, p = 0.035). In the RL group, right-handed male twins (M = 0.288, SE = 0.073) showed a higher AI_vol_ than their left-handed co-twins (M = 0.068, SE = 0.102, p = 0.043), whereas the female RL twin pairs did not significantly differ (for right-handers, M = 0.140, SE = 0.054; for left-handers, M = 0.225, SE = 0.076, p = 0.277).

### Cerebral Asymmetry Effects

Handedness is only weakly related to cerebral asymmetry for language, so we next examined the effect of LI values on asymmetry of the arcuate fasciculus. We sorted the twins so that the first twin in each pair had the higher LI, indicating stronger left-cerebral asymmetry for language. They were then allocated to two groups, one (24 twin pairs) in which the pairs were concordant for left-cerebral asymmetry, the other (7 twin pairs) in which one showed typical left-cerebral asymmetry and the other did not (LI <0.1). One pair was excluded because both members showed atypical language dominance. In the concordant group, LIs ranged from 0.51 to 0.96 (mean = 0.861) for twin A (the more lateralized) and from 0.14 to 0.93 (mean = 0.668) for twin B (the less lateralized). In the discordant group the ranges were 0.18 to 0.99 (mean = 0.716) for twin A and 0.09 to -0.93 (mean = −0.377) for twin B.

**Figure 3:**
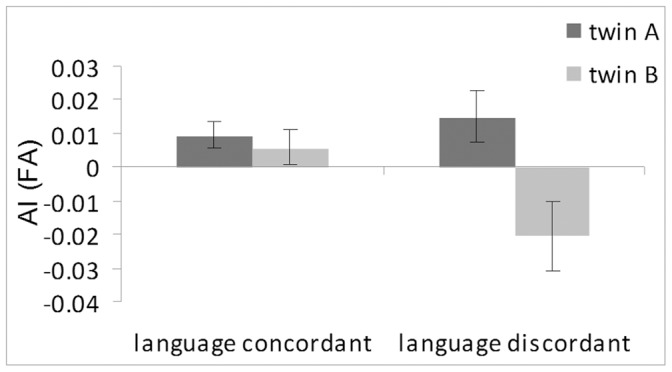
Cerebral language dominance. Asymmetry indices (AI) for the FA values of the arcuate fasciculus according to cerebral language dominance are shown. Twin pairs with discordant language dominance showed reversed AI_FA_ asymmetry.

Analysis of variance, with concordance and gender as between-twin factors, showed a positive AI_FA_ (mean = 0.0123) in the more left-lateralized twin and negative AI_FA_ (mean = −0.0074) in the less left-lateralized twin (F_1,27_ = 8.17, p = 0.008). A significant interaction revealed reversal of AI_FA_ to be restricted to the discordant group (F_1,27_ = 5.373, p = 0.028) with the left-cerebrally dominant twin showing leftward (M = 0.015, SE = 0.008) and the right-cerebrally dominant twins rightward (M = −0.021, SE = 0.010) asymmetry (see Figure 3).

A similar trend was also observed for AI_MD_ (F_(1,27) = _3.485, p = 0.073) and AI_RD_ (F_(1,27)_ = 3.039, p = 0.093) with the more left-cerebral dominant twin showing lower MD and RD values (MD, M = −0.005, SE = 0.005; RD, M = −0.004, SE = 0.004) than the less left-cerebral dominant one (MD, M = 0.004, SE = 0.003; RD = 0.004, SE = 0.004). An interaction revealed that reversals of the asymmetry were restricted to the male twin pairs discordant for language dominance with the left-cerebral dominant twins showing rightward asymmetry in MD (M = −0.004, SE = 0.014) and RD (M = −0.005, SE = 0.014), and the right-cerebral dominant ones showing leftward asymmetry of MD (M = 0.030, SE = 0.010) and RD (M = 0.029, SE = 0.010). Furthermore in the twin pairs with opposite language dominance, the left-cerebrally dominant twin showed significantly lower AI_RD_ values on the 2% threshold (M = −0.008, SE = 0.009) than his right-cerebrally dominant co-twin (M = 0.021, SE = 0.008; F_(1,27)_ = 5.067, p = 0.033), whereas the effect did not reach significance on the 1% threshold, probably reflecting more pronounced asymmetry of myelination in the core area of the tract. This effect was irrespective of gender.

Analysis of volume asymmetry and AD asymmetry revealed no significant effects of either language dominance or gender and no significant effect on the number of words generated in the Word Generation Task was observed.

## Discussion

The effect of handedness on arcuate fasciculus asymmetry was evident only in males, but not in females, with right-handed male twin pairs showing a stronger asymmetry in the FA and MD values than those with discordant handedness, and the left-handed males of the RL group showing a diminished volume asymmetry. This result accords with another study of male monozygotic twins, in which right-handed pairs showed asymmetries of lobar volumes while pairs discordant for handedness showed loss of asymmetry, especially in the temporal lobe [37]. In that study, moreover, the genetic control over the lobar volumes was twice as high in the right-handed pairs as in the discordant pairs. These results fit moderately well with genetic theories of handedness and brain asymmetries, assuming a lateralizing gene that introduces a bias toward right-handedness and left cerebral dominance for language, whereas in absence of the gene asymmetries develop as a matter of chance [11], [16]. This lack of bias has been attributed either to an allele that does not express the bias [38], or to epigenetic cancelling of the gene [39].

Nevertheless we found no effect of handedness in the female twins. Effects of gender on brain asymmetry have been reported before, with the majority of studies showing more pronounced anatomical asymmetries in males [40]. For example, in a meta-analysis reduced planum temporale asymmetry in left-handers and females was reported [41] and white matter asymmetries in frontal and temporal regions were found to be more pronounced in males [42]. Furthermore, right-handed males exhibited a leftward connectivity of the arcuate fasciculus which was absent in left-handed males whereas females showed a bilateral distribution irrespective of handedness [43]. The more symmetrical connectivity of the arcuate fasciculus in females has been replicated in some [34], [35] but not all studies [44], [45].

Handedness is at best an indirect measure of cerebral asymmetry for language, and the effects of cerebral asymmetry for language, as derived from fMRI, showed more clear-cut results, at least with respect to fractional anisotropy of the arcuate fasciculus. Left-cerebrally dominant twin pairs showed higher FA on the left, while twin pairs with discordant language asymmetry showed reversal, with the left-cerebrally dominant twins showing leftward asymmetry of the FA and the right-cerebrally dominant co-twins showing rightward asymmetry. These effects were independent of gender. Fractional anisotropy reflects the directionality of the diffusion processes and is influenced by axonal packing, alignment, and diameter [6]. Thus asymmetry of language representation was directly mapped onto structural asymmetry of the anisotropic diffusion in the arcuate fasciculus. This influence may be nongenetic, and due to neural potentiation resulting from activation of the language circuits.

On the other hand, reversals of the anatomical asymmetry index for mean diffusivity and radial diffusivity were only evident in male but not in female twin pairs with discordant language dominance. Mean diffusivity indicates the mean overall diffusion and is influenced by diffusion barriers such as cell membranes or myelin sheath. Axial and radial diffusivity describe the diffusion parallel and perpendicular to the principal axis, respectively. Radial diffusivity has been linked to the amount of myelin in a tissue. For example, mice lacking myelin in the CNS were found to show increased RD but unchanged AD values compared to age-matched control mice [46]. Our results might therefore indicate that the tracts in the dominant hemisphere are more heavily myelinated, at least in men. It is important to note though, that although each diffusion parameter emphasizes at least partially distinct microstructural properties of white matter pathways, none of them is determined entirely by one structural element [47].

High anisotropic diffusion in the hemisphere dominant for language might reflect rapid transmission of linguistic information [9], given the involvement of the arcuate fasciculus in phonologic [32], [48], [49] and syntactic processing [2]. Reduced FA values in the left arcuate fasciculus are also associated with deficits in syntactic processing in patients with primary progressive aphasia [50] and after left-hemispheric stroke [51]. The reversal of FA asymmetry in those with atypical language dominance is also consistent with emerging evidence that the usual left-hemisphere structures associated with language are reversed in some individuals [52], [53], [54].

Other activity may also alter connectivity; for example, musicians have larger tract volume and higher FA values in the left and right arcuate fasciculus than non-musicians [55]. The effects of environmentally-induced plasticity are also likely to accumulate with age, reducing heritability. In a large scale study investigating over 700 twins and their siblings, heritability of the FA values dropped from 70–80% in adolescents to only 30–40% in adults [56], due to influences of sex, age, intellectual performance and socio-economic status.

Although our results suggest a nongenetic influence, they can also be considered consistent with the genetic model outlined above. The majority of twin pairs concordant for left-cerebral language dominance may well inherit the gene biasing cerebral asymmetry for language to the left, while discordant pairs lack this influence so that both cerebral dominance and arcuate fasciculus asymmetry are matters of chance. Of course some twins lacking the gene may also fall by chance in the concordant group, although most of the twins in that group may be assumed to inherit the lateralizing gene. Indeed, the results for cerebral asymmetry fit the genetic model better than those for handedness, supporting Annett’s conjecture [57] that the gene is primarily a cerebral asymmetry gene, with only indirect effects on handedness. Indeed, although some 22% of MZ twins are of opposite handedness [58], some 70% of them both show left-cerebral asymmetry for language [12], illustrating the relatively poor correspondence between handedness and cerebral asymmetry for language.

In summary, our results show a strong correspondence between cerebral asymmetry for language and asymmetry in connectivity within the arcuate fasciculus in monozygotic twins. They also show weaker evidence for an effect of handedness, although this was restricted to male twins. The fact that asymmetry of the arcuate fasciculus would be reversed between twins sharing the same genotype suggests a strong nongenetic influence, although the data are also consistent with a genetic model in which lack of a lateralizing gene leaves the direction of asymmetry open to chance.
